# Sex-Specific Safety Signals of Finerenone Versus Spironolactone: Insights From the FDA Adverse Event Reporting System

**DOI:** 10.7759/cureus.113379

**Published:** 2026-07-25

**Authors:** Abhinandan Singla, Gurpreet Singh, Gurnoor Kaur, Shivansh Luthra, Chirag Lodha, Mashaal S Malik, Sidra Kalsoom, Mohammed S Alo, Syed S Ali, Muhammad Zulqarnain

**Affiliations:** 1 Internal Medicine, Mercy Health St. Vincent Medical Center, Toledo, USA; 2 Medicine, Government Medical College Amritsar, Amritsar, IND; 3 Internal Medicine, University of South Florida Morsani College of Medicine, Tampa, USA; 4 Internal Medicine, Aga Khan University, Karachi, PAK; 5 Internal Medicine, Conemaugh Memorial Medical Center, Johnstown, USA; 6 Cardiology, Mercy Health St. Vincent Medical Center, Toledo, USA; 7 Interventional Cardiology, Mercy Health St. Vincent Medical Center, Toledo, USA

**Keywords:** adverse drug reactions, disproportionality analysis, faers, finerenone, mineralocorticoid receptor antagonist, pharmacovigilance, spironolactone, women

## Abstract

Background: Spironolactone, a non-selective mineralocorticoid receptor antagonist (MRA), frequently causes off-target hormonal effects in women. Finerenone, a selective non-steroidal MRA, may mitigate these effects, but real-world comparative data remain scarce.

Objective: This study aimed to compare adverse event reporting profiles of finerenone and spironolactone in female patients using the FDA Adverse Event Reporting System (FAERS).

Methods: We conducted a retrospective disproportionality analysis using FAERS data (January 2022-March 2026), identifying female primary suspect reports for finerenone (N = 715) and spironolactone (N = 4,651). Reporting odds ratios (ROR) with 95% confidence intervals were calculated across reproductive, endocrine, skin, and psychiatric domains. Hyperkalemia served as a positive control.

Results: Finerenone reporters were significantly older than spironolactone reporters (350 (75.9%) vs. 2,329 (59.9%) aged 65 years and older; p < 0.0001). Spironolactone demonstrated disproportionately higher reporting across all assessed domains compared to finerenone: reproductive (ROR 0.15, 95% CI 0.06-0.39), endocrine (ROR 0.20, 95% CI 0.07-0.58), psychiatric (ROR 0.26, 95% CI 0.16-0.43), and skin disorders (ROR 0.45, 95% CI 0.32-0.65). Hyperkalemia showed no directional signal (ROR 1.12, 95% CI 0.87-1.45). Notable individual spironolactone signals included polycystic ovaries, hypothyroidism, depression, and confusion.

Conclusions: Spironolactone generates substantially more spontaneous reports of hormonal, dermatological, and psychiatric adverse events in women than finerenone. While indication confounding and age differences limit direct causal inference, these reporting patterns align with finerenone's enhanced receptor selectivity. The persistent psychiatric signal for spironolactone warrants further investigation.

## Introduction

Mineralocorticoid receptor antagonists (MRAs) have occupied a central role in the management of heart failure and chronic kidney disease for several decades. Spironolactone, introduced in the 1960s, reduces all-cause mortality in patients with heart failure with reduced ejection fraction [1,2] and slows the progression of proteinuric kidney disease [3]. However, its clinical utility has always been tempered by a well-recognized constellation of hormonal side effects. The drug acts not only at mineralocorticoid receptors but also at androgen and progesterone receptors, producing anti-androgenic effects that manifest as menstrual irregularities, breast tenderness, reduced libido, and mood changes [4-6] - effects that are not trivial and contribute meaningfully to treatment discontinuation, particularly in younger women.

The significance of this tolerability problem has been underappreciated in clinical trials, largely because women have historically been under-enrolled in cardiovascular and nephrology outcome studies. Furthermore, it is increasingly recognized that women generally experience a higher burden of adverse drug reactions compared to men, driven by sex-based differences in pharmacokinetics and pharmacodynamics [7,8]. As the therapeutic indications for MRAs have expanded and prescribing has broadened to include women of reproductive age, not only for cardiac and renal disease but also for acne, hirsutism, and polycystic ovary syndrome, the tolerability gap has become increasingly clinically relevant.

Finerenone was designed to address this limitation. As a non-steroidal, highly selective MRA, it was developed with a structural profile that avoids off-target binding to sex hormone receptors, which is responsible for spironolactone's hormonal adverse effects [4]. The FIDELIO-DKD [9] and FIGARO-DKD [10] trials, along with pooled analyses [11,12], demonstrated that finerenone reduces cardiovascular and renal endpoints in patients with type 2 diabetes and chronic kidney disease. However, neither trial was designed to systematically assess hormonal tolerability, and neither enrolled populations that would allow meaningful comparison of sex hormone-related adverse events against an active spironolactone comparator.

Real-world pharmacovigilance data offer a complementary lens. The FDA Adverse Event Reporting System (FAERS) captures spontaneous reports across the full breadth of a drug's use in clinical practice, including adverse events that trials may not capture because they are too common to be considered noteworthy, too subjective to be systematically collected, or reported predominantly by patients rather than investigators [13-15]. To our knowledge, no published study has directly compared the adverse event reporting profiles of finerenone and spironolactone in women using FAERS data. We therefore conducted this analysis to characterize and compare sex-specific adverse event signals across hormonal, dermatological, and psychiatric domains in female reporters, using disproportionality methodology.

## Materials and methods

Study design and data source 

This was a retrospective disproportionality analysis using spontaneous adverse event reports from the FAERS [13,15]. FAERS is a publicly available post-marketing surveillance database maintained by the Food and Drug Administration (FDA) that receives mandatory reports from manufacturers and voluntary reports from healthcare professionals and consumers. Quarterly ASCII data files were downloaded for the period of January 2022 through March 2026 (2022Q1-2026Q1). This start date was selected to align the study window with the post-approval surveillance period of finerenone, which received FDA approval in August 2021.

Case selection 

To provide a detailed framework for the analysis, explicit criteria were established for the data extraction.

Inclusion Criteria

Reports were included if they met the following criteria: (1) finerenone (trade name Kerendia) or spironolactone appeared as the primary suspect (PS) or secondary suspect (SS) drug, searching both generic and proprietary names to capture variant entries; (2) patient sex was explicitly coded as female; and (3) patient age was 18 years or older. 

Exclusion Criteria

Reports were excluded based on the following criteria: (1) either drug appeared only in a concomitant or drug interaction role; (2) sex was coded as male, missing, unknown, or ambiguous; (3) pediatric reports (<18 years) or reports with missing/unspecified age; (4) both spironolactone and finerenone were co-administered or listed sequentially in the same report to prevent cross-contamination of adverse event attribution; and (5) duplicate reports, which were identified and removed by analyzing unique case numbers, keeping only the most recent version of the report.

Adverse event selection 

Adverse events of interest were identified a priori across four MedDRA (Medical Dictionary for Regulatory Activities, version 29.0, International Council for Harmonisation of Technical Requirements for Pharmaceuticals for Human Use (ICH)) system organ classes: reproductive system and breast disorders, endocrine disorders, skin and subcutaneous tissue disorders, and psychiatric disorders. Within each class, individual preferred terms were selected based on biological plausibility for mineralocorticoid receptor antagonist-related effects [4-6], with particular attention to mechanisms likely to differ between selective and non-selective MRAs. All term selections were made before data extraction and were not modified after viewing the data.

Disproportionality analysis 

For each drug-adverse event pair, a standard 2 x 2 contingency table was constructed, comparing the count of reports of the adverse event of interest for the drug of interest against all other adverse events and against all other drugs in the database. The reporting odds ratio (ROR) was used as the primary measure of disproportionate reporting. A continuity correction of 0.5 was added to each cell of the 2 x 2 table to allow calculation when one or more cells contained zero counts. Ninety-five percent confidence intervals were derived using the standard logarithmic approximation. A pharmacovigilance signal was defined as an ROR lower 95% confidence bound exceeding 1.0.

A primary domain-level analysis was conducted by pooling all preferred terms within each system organ class into a single grouped estimate. Individual preferred term-level results are reported as a secondary analysis. Terms with fewer than three reports in both cohorts were not formally interpreted as signals.

Positive control 

Hyperkalemia was selected as a positive control because it represents an expected, on-target pharmacological effect shared by all MRAs, regardless of receptor selectivity. Under the assumption that both drugs carry equivalent risk of hyperkalemia relative to other drugs in FAERS, the ROR for this term was expected to approach 1.0 in both cohorts with no directional signal. Confirmation of this expectation was used as a methodological validity check.

Age distribution 

Age at the time of reporting was extracted from the demographic file and categorized as under 65 years or 65 years and older. This threshold was selected to stratify the population by age, acknowledging that available FAERS data do not include menstrual or menopausal status.

Sensitivity analysis 

A sensitivity analysis restricting spironolactone cases to those with cardiac or renal indications-thereby excluding cases attributable to dermatological and endocrine prescribing in younger women-was planned but could not be performed due to incompletely coded indication data in FAERS. The age distribution comparison was used as a quantitative proxy to characterize the likely magnitude and direction of indication confounding.

Statistical analysis, software, and ethics 

Data manipulation and statistical analyses were performed using Python version 3.12 (Python Software Foundation), utilizing the pandas library for data processing and SciPy (version 1.11) for statistical testing. Statistical significance of the disproportionality was assessed using the chi-square test with Yates' continuity correction. Fisher's exact test was utilized when expected cell counts in the 2 x 2 table were less than 5. Continuous variables were compared using independent samples t-tests, and categorical demographic variables were compared using chi-square tests. Statistical significance was set at a two-sided alpha of 0.05. As this study analyzed publicly available, de-identified post-marketing surveillance data, institutional review board approval was not required.

## Results

Study population and demographics 

After deduplication and sex restriction, 5,366 female primary suspect reports were available for analysis: 4,651 for spironolactone and 715 for finerenone. The smaller finerenone cohort reflects its narrower approved indication, a more restricted prescribing population, and a shorter post-marketing surveillance period.

The demographic characteristics are presented in Table 1. Spironolactone reporters in the FAERS database were substantially younger than finerenone reporters. Among those with reported ages, 1,559 (40.1%) of spironolactone reporters were under the age of 65, compared with 111 (24.1%) for finerenone (p < 0.0001). The practical implication is that spironolactone reporters are substantially younger than finerenone reporters, which likely correlates with different primary prescribing indications.

**Table 1:** Demographic characteristics of female FAERS reporters for spironolactone and finerenone. - indicates empty or non-applicable cells. A p-value is considered statistically significant at a threshold of p < 0.05, and significant values are denoted by *. The test statistic represents the chi-square numerical value. The statistical test used to calculate the p-value for the age distribution comparison was the chi-square test with Yates' continuity correction. FAERS: FDA Adverse Event Reporting System

Characteristic	Spironolactone (N = 4,651)	Finerenone (N = 715)	Test statistic (chi-square)	P-value
Age reporting status	-	-	-	-
Unspecified age data	763 (16.4%)	254 (35.5%)	-	-
Age distribution (evaluable reports)	-	-	-	-
Age < 65 years	1,559 (40.1%)	111 (24.1%)	44.04	< 0.0001*
Age ≥ 65 years	2,329 (59.9%)	350 (75.9%)	-	-

Positive control: hyperkalemia 

Hyperkalemia, used as a methodological positive control, showed an ROR of 1.12 (95% CI 0.87-1.45), with no disproportionate reporting in either direction. This is the expected result for a class effect shared equally by both drugs relative to the broader FAERS comparator population [16], and it supports the validity of the analytical approach. The absence of a general reporting bias favoring either drug confirms that the directional signals observed in hormonal and psychiatric domains reflect genuine differences in adverse event reporting patterns rather than a systematic database artifact.

Domain-level analysis 

**Figure 1:**
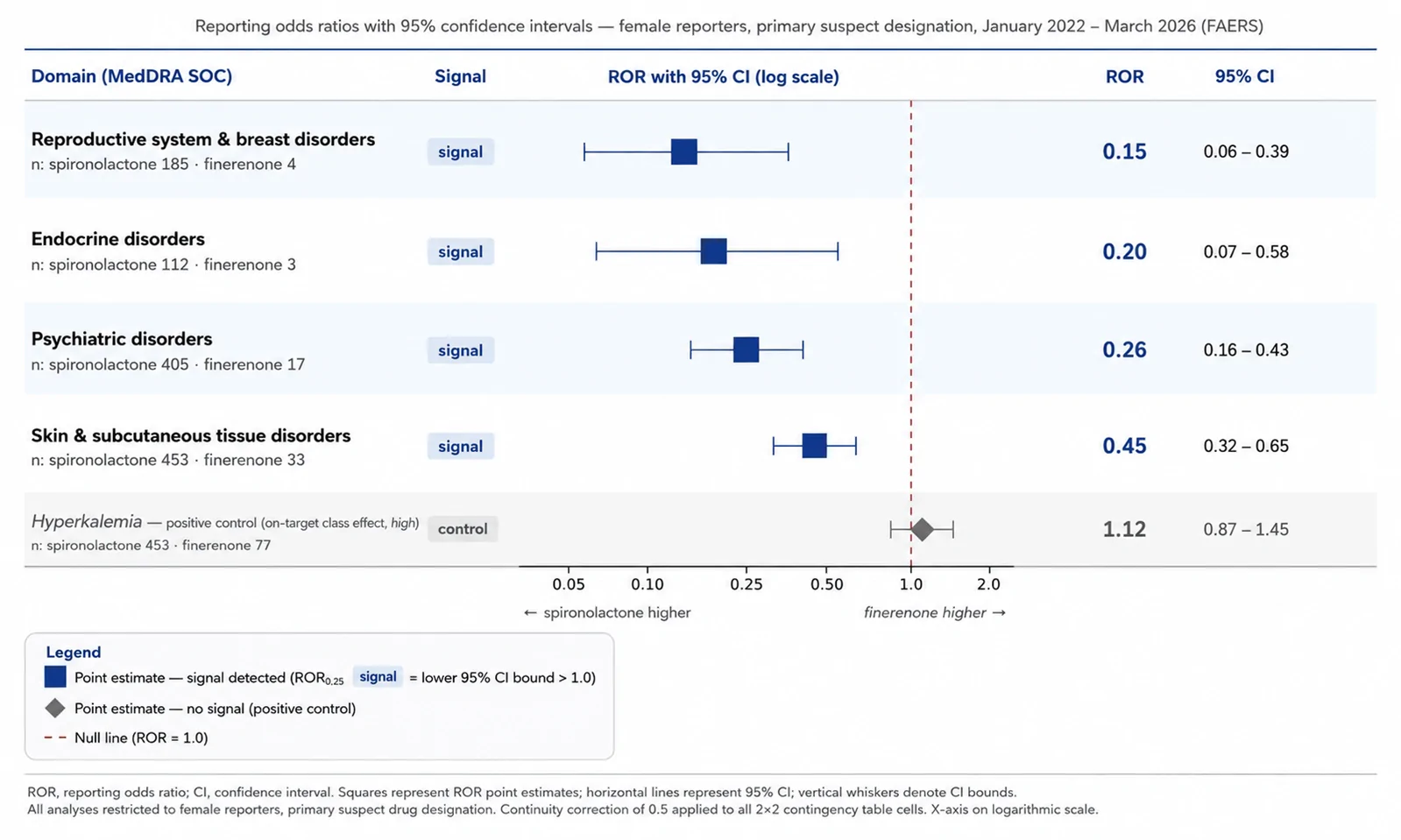
Forest plot of reporting odds ratios (RORs) comparing adverse event signals between finerenone and spironolactone in female reporters. Squares represent point estimates; horizontal lines represent 95% CI. All analyses restricted to female reporters, primary suspect designation, January 2022–March 2026. ROR < 1.0 indicates disproportionately higher reporting for spironolactone. Hyperkalemia was included as a positive control (diamond). The dashed vertical line denotes null (ROR = 1.0). ROR: reporting odds ratio The data manipulation and statistical analyses used to generate our results were performed using Python version 3.12 (Python Software Foundation).   Specifically, we utilized the pandas library for data processing and SciPy (version 1.11) for statistical testing.

The primary domain-level pooled analysis demonstrated significantly lower reporting for finerenone compared to spironolactone across all four adverse event domains examined (Table 2). The strongest signal disparity was seen in the reproductive system and breast disorders domain, where spironolactone generated over sixfold more proportional reporting than finerenone (ROR 0.15, 95% CI 0.06-0.39). A similarly pronounced difference was observed in endocrine disorders (ROR 0.20, 95% CI 0.07-0.58) and psychiatric disorders (ROR 0.26, 95% CI 0.16-0.43). The skin domain showed a somewhat attenuated but still statistically significant disparity (ROR 0.45, 95% CI 0.32-0.65), driven in part by large spironolactone case counts for pruritus and rash. Figure 1 shows the forest plot for all four domains examined.

**Table 2:** Domain-level disproportionality analysis (primary analysis). Signal is defined as the upper bound of the 95% CI < 1.0 (indicating significantly lower reporting for finerenone relative to spironolactone). A continuity correction of 0.5 was applied to all 2 x 2 cells. A p-value is considered statistically significant at a threshold of p < 0.05, and significant values are denoted by *. The test statistic represents the chi-square numerical value. P-values were calculated using the chi-square test with Yates' continuity correction. All analyses were restricted to female reporters, primary suspect designation, January 2022–March 2026. ROR: reporting odds ratio

Domain (MedDRA SOC)	Spironolactone (n)	Finerenone (n)	ROR	95% CI	Test statistic (chi-square)	P-value	Signal detected
Reproductive system and breast disorders	185	4	0.15	0.06-0.39	20.30	<0.0001*	Yes
Endocrine disorders	112	3	0.20	0.07-0.58	11.20	<0.001*	Yes
Skin and subcutaneous tissue disorders	453	33	0.45	0.32-0.65	17.00	<0.0001*	Yes
Psychiatric disorders	405	17	0.26	0.16-0.43	30.50	<0.0001*	Yes
Hyperkalemia (positive control)	453	77	1.12	0.87-1.45	0.50	0.479	No

Preferred term-level analysis 

At the individual preferred term level (Table 3), statistically significantly lower reporting for finerenone was observed for eight terms: polycystic ovaries (ROR 0.03, 95% CI 0.00-0.55), hypothyroidism (ROR 0.14, 95% CI 0.04-0.51), depression (ROR 0.11, 95% CI 0.02-0.56), anxiety (ROR 0.31, 95% CI 0.11-0.92), insomnia (ROR 0.35, 95% CI 0.15-0.82), confusional state (ROR 0.37, 95% CI 0.14-0.97), rash (ROR 0.43, 95% CI 0.21-0.86), and pruritus (ROR 0.54, 95% CI 0.30-0.97). No individual term showed a statistically significant signal in the direction of finerenone.

**Table 3:** Preferred term-level disproportionality analysis Signal (Yes) denotes disproportionately higher spironolactone reporting compared to finerenone. Insuff. n: fewer than three reports in both cohorts—ROR and tests not formally interpreted. - indicates empty or non-applicable cells. A p-value is considered statistically significant at a threshold of p < 0.05, and significant values are denoted by *. The test statistic represents the chi-square numerical value. P-values were calculated using the chi-square test with Yates' continuity correction (or Fisher's exact test where expected cell counts were < 5). A continuity correction of 0.5 was applied to all cells.

MedDRA preferred term	Spiro (n)	Fin (n)	ROR	95% CI	Test statistic (chi-square)	P-value	Signal
Reproductive system and breast disorders	-	-	-	-	-	-	-
Polycystic ovaries	93	0	0.03	0.00–0.55	13.10	< 0.001*	Yes
Breast pain	8	3	2.68	0.77–9.34	0.81	0.368	No
Amenorrhea	10	0	0.31	0.02–5.28	-	-	No
Menstrual irregularities	8	0	0.38	0.02–6.62	-	-	No
Breast tenderness	5	0	0.59	0.03–10.69	-	-	No
Vulvovaginal dryness	5	0	0.59	0.03–10.69	-	-	No
Female sexual dysfunction	4	0	0.72	0.04–13.42	-	-	No
Dyspareunia	2	0	-	-	-	-	Insuff. n
Breast discharge	1	1	6.51	0.68–62.66	-	-	Insuff. n
Endocrine disorders	-	-	-	-	-	-	-
Hypothyroidism	110	2	0.14	0.04–0.51	10.30	0.001*	Yes
Thyroid mass	2	1	3.90	0.51–29.60	-	-	Insuff. n
Skin and subcutaneous tissue disorders	-	-	-	-	-	-	-
Pruritus	147	12	0.54	0.30–0.97	4.00	0.045*	Yes
Rash	126	8	0.43	0.21–0.86	4.90	0.027*	Yes
Alopecia	54	3	0.41	0.14–1.22	0.65	0.420	No
Urticaria	38	7	1.27	0.58–2.78	0.15	0.698	No
Stevens-Johnson syndrome	26	0	0.12	0.01–2.00	3.65	0.056	No
Acne	18	0	0.18	0.01–2.91	2.50	0.114	No
Skin hyperpigmentation	6	2	2.50	0.58–10.79	-	-	No
Hyperhidrosis	33	1	0.29	0.06–1.49	3.20	0.073	No
Psychiatric disorders	-	-	-	-	-	-	-
Depression	85	1	0.11	0.02–0.56	8.70	0.003*	Yes
Insomnia	101	5	0.35	0.15–0.82	5.20	0.022*	Yes
Anxiety	71	3	0.31	0.11–0.92	4.10	0.042*	Yes
Confusional state	77	4	0.37	0.14–0.97	4.20	0.040*	Yes
Suicidal ideation	19	1	0.50	0.09–2.63	0.15	0.698	No
Completed suicide	27	0	0.12	0.01–1.93	3.80	0.051	No
Panic reaction	9	1	1.03	0.18–5.75	-	-	No
Hallucination	16	2	0.98	0.26–3.73	-	-	No

Several clinically relevant terms showed a complete absence of finerenone reports alongside meaningful spironolactone counts, including acne (spironolactone N = 18, finerenone N = 0), amenorrhea (N = 10 vs. N = 0), Stevens-Johnson syndrome (N = 26 vs. N = 0), completed suicide (N = 27 vs. N = 0), and a range of menstrual and breast-related terms. These observations cannot be formally tested for disproportionality but are reported descriptively due to their potential clinical significance. Full preferred term-level results are shown in Table 3.

## Discussion

This pharmacovigilance analysis offers the first systematic comparison of adverse-event reporting profiles for finerenone and spironolactone in women, drawing on post-marketing FAERS data [13] over a four-year surveillance window. The central finding is straightforward: spironolactone generates substantially more spontaneous adverse event reporting than finerenone across every hormonal and systemic domain examined, with statistically significant domain-level signals for reproductive, endocrine, psychiatric, and dermatological adverse events. The positive control performed as expected, confirming that this divergence is not attributable to a general database artifact. Taken together, these findings are consistent with the hypothesis that finerenone's structural selectivity for the mineralocorticoid receptor translates into a meaningfully different real-world adverse event reporting profile in women [4].

The magnitude of the reproductive system disparity is striking. The domain-level ROR of 0.15 indicates that reproductive and breast adverse events are reported at approximately one-seventh the proportional rate for finerenone compared to spironolactone, and the polycystic ovary signal alone (ROR 0.03) reflects an almost complete absence of this finding in the finerenone cohort. This is broadly consistent with the mechanistic explanation: spironolactone's anti-androgenic activity, mediated through androgen receptor blockade and inhibition of testosterone synthesis, accounts for its long-documented effects on menstrual function, ovarian morphology, and breast tissue [4-6]. Finerenone does not bind androgen or progesterone receptors at clinically relevant concentrations [4], and the near-absence of these reports in FAERS aligns with what its pharmacological profile would predict.

However, this interpretation requires caution because the two cohorts differ in a way that independently accounts for much of the reproductive difference. Spironolactone reporters in FAERS are substantially younger than finerenone reporters; 1559 (40.1%) of known-age spironolactone reporters were under 65, compared with 111 (24.1%) for finerenone, a difference that was highly statistically significant. Spironolactone is prescribed not only for heart failure [1,2] and chronic kidney disease [3] but also for acne, hirsutism, and polycystic ovary syndrome. These indications concentrate on use in younger women who are biologically more likely to experience menstrual and ovarian adverse effects. Finerenone's approved indication is restricted to diabetic chronic kidney disease [9-12], a condition with a prevalence weighted toward older, postmenopausal women. The absence of menstrual adverse events in the finerenone cohort may therefore partly or substantially reflect the absence of menstruating women among its reporters, rather than a direct drug effect. We were unable to conduct a formal indication-restricted sensitivity analysis due to incomplete coding of indication data in FAERS, and this remains the principal limitation of the reproductive findings.

The psychiatric findings are more difficult to explain by demographics alone and, in some respects, are the most interesting result of this analysis. Spironolactone showed significantly lower-bound signals for depression (ROR 0.11), anxiety (ROR 0.31), insomnia (ROR 0.35), and confusional state (ROR 0.37), all driven by substantial spironolactone case counts relative to finerenone.

The critical observation is that finerenone reporters were older, a population that might be expected to carry a higher background burden of depression, cognitive impairment, and sleep disturbance, given the age-related prevalence of these conditions. The fact that spironolactone nonetheless dominates these signals despite a comparatively younger reporting population suggests that the psychiatric disparity is not simply an age artifact and may reflect genuine pharmacological differences between the drugs.

Spironolactone has been previously reported to interact with progesterone receptors in the central nervous system, and animal models have demonstrated neurosteroid-like effects on GABAergic signaling that could plausibly underlie mood and cognitive effects [4,5]. Whether this mechanism accounts for the clinical signal seen in FAERS is unknown. Still, the consistency of the psychiatric domain findings across multiple distinct preferred terms-depression, anxiety, insomnia, and confusional state-lends them a degree of biological coherence that makes dismissal as noise difficult to justify.

The finding of hypothyroidism was not anticipated and warrants separate comment. Spironolactone showed a statistically significant lower-bound signal for hypothyroidism (ROR 0.14, 95% CI 0.04-0.51), with 110 reports, compared with 2 for finerenone. The mechanism is not obvious: spironolactone neither inhibits thyroid hormone synthesis nor acts at thyroid receptors. One possible explanation is that hypothyroidism is more prevalent in the spironolactone population because of the age and indication overlap; women being treated for heart failure or hypertension with spironolactone may coincidentally carry a diagnosis of hypothyroidism that is then captured in FAERS [13] as a co-reported event rather than a drug-caused event. A second possibility is that the sex hormone-binding globulin elevation known to occur with spironolactone treatment [4,5] alters thyroid hormone transport and equilibrium in ways that generate or unmask subclinical hypothyroidism. This signal is hypothesis-generating and should not be over-interpreted from an FAERS dataset, but it is sufficiently unexpected to warrant notation and prospective examination.

The dermatological findings reflect a mixture of signals. Pruritus and rash showed significantly lower-bound signals for spironolactone (RORs of 0.54 and 0.43, respectively), which may reflect true pharmacological differences or, in part, the greater case count and therefore statistical power in the spironolactone cohort. Notably, acne and Stevens-Johnson syndrome were reported exclusively in spironolactone cases. The absence of acne reports for finerenone is consistent with its approved indication [9], which states that patients with diabetic kidney disease are unlikely to report acne as an adverse event regardless of drug exposure. It underscores the difficulty of comparing drugs with fundamentally different prescribing contexts within FAERS.

Strengths and limitations 

The principal strength of this analysis is its novelty. No prior publication has directly compared the adverse event profiles of finerenone and spironolactone in women using FAERS data, and this question has practical clinical relevance given the expanding therapeutic landscape for MRAs. The use of a positive control, the application of domain-level pooling to address low finerenone case counts, and the explicit quantification of age distribution confounding all represent methodological choices that strengthen the interpretive framework.

The limitations are substantial and must be prominently acknowledged. FAERS is a spontaneous reporting system subject to well-documented biases, including underreporting, notoriety bias (where well-known adverse effects of established drugs are more readily reported), and stimulated reporting following media attention [14,17]. The Weber effect [17], the tendency for adverse event reporting rates to peak in the years immediately following drug approval and decline thereafter, may disproportionately suppress finerenone signals, given its shorter post-marketing history. However, this would be expected to bias toward finding fewer finerenone signals rather than creating false spironolactone signals. Critically, FAERS provides no denominators [13], precluding the calculation of true incidence rates or formal adjusted risk estimates. The indication-confounding problem detailed above is not fully resolvable with the available data. Finally, the analysis is restricted to female reporters as coded in FAERS, and miscoding of sex, particularly for reports submitted by manufacturers reconstructing cases from secondary sources, cannot be excluded. These limitations collectively mean that this analysis should be interpreted as hypothesis-generating rather than confirmatory. The findings are consistent with finerenone's mechanistic selectivity and provide a structured characterization of the FAERS signal landscape. Still, they do not establish causation, and they cannot quantify absolute risk differences between the two drugs in any clinical population.

Clinical implications 

Despite the limitations inherent in pharmacovigilance methodology, these findings carry practical relevance. Women with chronic diabetic kidney disease or heart failure who are candidates for MRA therapy represent a population in whom tolerability is a genuine clinical consideration. Premenopausal women in particular face a set of adverse effects with spironolactone-i.e., menstrual disruption, breast symptoms, and reduced libido-that are not trivial and that contribute to real-world discontinuation rates that trial data may not fully capture [1,3]. The near-complete absence of these signals in the finerenone FAERS dataset, while not proof of superiority, is at minimum consistent with the mechanistic expectation that a receptor-selective agent would avoid the off-target effects of an older, non-selective drug [4]. For clinicians weighing MRA options in women where tolerability matters, particularly younger women with diabetic kidney disease, these data provide supportive, if preliminary, evidence that finerenone's hormonal adverse event reporting burden may be meaningfully lower than spironolactone's in real-world practice.

## Conclusions

In this pharmacovigilance analysis of FAERS data from 2022 to 2026, spironolactone generated substantially more spontaneous adverse event reports than finerenone across reproductive, endocrine, psychiatric, and dermatological domains among female reporters. Domain-level signals were statistically significant in all four categories, and individual signals for polycystic ovaries, hypothyroidism, depression, anxiety, insomnia, and confusional state were prominent. The expected null result for hyperkalemia confirmed the methodological validity of the approach. Interpretation is constrained by significant age-distribution differences between cohorts and by the limitations inherent in spontaneous reporting data. Still, the pattern of findings is consistent with the mechanistic selectivity of finerenone for the mineralocorticoid receptor. These results support the conduct of prospective studies powered to evaluate hormonal and psychiatric tolerability as endpoints in women receiving MRA therapy and provide a structured evidentiary foundation for clinical discussions regarding drug selection in female patients for whom tolerability is a priority.

## References

[1] Pitt B, Zannad F, Remme WJ (1999). The effect of spironolactone on morbidity and mortality in patients with severe heart failure. Randomized Aldactone Evaluation Study Investigators. N Engl J Med.

[2] Pitt B, Pfeffer MA, Assmann SF (2014). Spironolactone for heart failure with preserved ejection fraction. N Engl J Med.

[3] Zannad F, McMurray JJ, Krum H (2011). Eplerenone in patients with systolic heart failure and mild symptoms. N Engl J Med.

[4] Kolkhof P, Bärfacker L (2017). 30 years of the mineralocorticoid receptor: mineralocorticoid receptor antagonists: 60 years of research and development. J Endocrinol.

[5] Wang Y, Lipner SR (2020). Retrospective analysis of adverse events with spironolactone in females reported to the United States Food and Drug Administration. Int J Womens Dermatol.

[6] Fareed J (2015). Antithrombotic therapy in 2014: making headway in anticoagulant and antiplatelet therapy. Nat Rev Cardiol.

[7] Zucker I, Prendergast BJ (2020). Sex differences in pharmacokinetics predict adverse drug reactions in women. Biol Sex Differ.

[8] Rademaker M (2001). Do women have more adverse drug reactions?. Am J Clin Dermatol.

[9] Bakris GL, Agarwal R, Anker SD (2020). Effect of finerenone on chronic kidney disease outcomes in type 2 diabetes. N Engl J Med.

[10] Filippatos G, Anker SD, Agarwal R (2021). Finerenone and cardiovascular outcomes in patients with chronic kidney disease and type 2 diabetes. Circulation.

[11] Agarwal R, Filippatos G, Pitt B (2022). Cardiovascular and kidney outcomes with finerenone in patients with type 2 diabetes and chronic kidney disease: the FIDELITY pooled analysis. Eur Heart J.

[12] Yang P, Cornelio RN, Reynoldson C (2022). Finerenone: a new non-steroidal mineralocorticoid receptor antagonist for the treatment of chronic kidney disease associated with type 2 diabetes. Ann Pharmacother.

[13] (2024). US Food and Drug Administration. FDA Adverse Event Reporting System (FAERS) Public Dashboard. https://fis.fda.gov/extensions/FPD-QDE-FAERS/FPD-QDE-FAERS.html.

[14] Bate A, Evans SJ (2009). Quantitative signal detection using spontaneous ADR reporting. Pharmacoepidemiol Drug Saf.

[15] Sakaeda T, Tamon A, Kadoyama K, Okuno Y (2013). Data mining of the public version of the FDA Adverse Event Reporting System. Int J Med Sci.

[16] van Puijenbroek EP, Bate A, Leufkens HG, Lindquist M, Orre R, Egberts AC (2002). A comparison of measures of disproportionality for signal detection in spontaneous reporting systems for adverse drug reactions. Pharmacoepidemiol Drug Saf.

[17] Hoffman KB, Dimbil M, Erdman CB, Tatonetti NP, Overstreet BM (2014). The Weber effect and the United States Food and Drug Administration's Adverse Event Reporting System (FAERS): analysis of sixty-two drugs approved from 2006 to 2010. Drug Saf.

